# Bromodomain and Extraterminal (BET) Protein Inhibition Restores Redox Balance and Inhibits Myofibroblast Activation

**DOI:** 10.1155/2019/1484736

**Published:** 2019-04-18

**Authors:** Carmel J. W. Stock, Charalambos Michaeloudes, Patricia Leoni, Andrew L. Durham, Sharon Mumby, Athol U. Wells, Kian Fan Chung, Ian M. Adcock, Elisabetta A. Renzoni, Gisela E. Lindahl

**Affiliations:** ^1^Interstitial Lung Disease Unit, Royal Brompton Hospital and National Heart and Lung Institute, Imperial College London, Sydney Street, London SW3 6NP, UK; ^2^Airway Disease Section, National Heart and Lung Institute, Imperial College London, London SW3 6LY, UK

## Abstract

**Background and Objective:**

Progressive pulmonary fibrosis is the main cause of death in patients with systemic sclerosis (SSc) with interstitial lung disease (ILD) and in those with idiopathic pulmonary fibrosis (IPF). Transforming growth factor-*β* (TGF-*β*) and NADPH oxidase- (NOX-) derived reactive oxygen species (ROS) are drivers of lung fibrosis. We aimed to determine the role of the epigenetic readers, bromodomain and extraterminal (BET) proteins in the regulation of redox balance in activated myofibroblasts.

**Methods:**

In TGF-*β*-stimulated fibroblasts, we investigated the effect of the BET inhibitor JQ1 on the mRNA expression of the prooxidant gene NOX4 and the antioxidant gene superoxide dismutase (SOD2) by quantitative RT-PCR, the antioxidant transcription factor NF-E2-related factor 2 (Nrf2) activity by a reporter assay, and intracellular ROS levels by dichlorofluorescein staining. Myofibroblast activation was determined by *α*-smooth muscle actin immunocytochemistry. The role of specific BET protein isoforms in NOX4 gene regulation was studied by siRNA silencing and chromatin-immunoprecipitation.

**Results and Conclusions:**

Affymetrix gene array analysis revealed increased* NOX4 *and reduced* SOD2 *expression in SSc and IPF fibroblasts.* SOD2* silencing in non-ILD control fibroblasts induced a profibrotic phenotype. TGF-*β* increased* NOX4* and inhibited* SOD2* expression, while increasing ROS production and myofibroblast differentiation. JQ1 reversed the TGF-*β*-mediated* NOX4*/*SOD2* imbalance and Nrf2 inactivation and attenuated ROS production and myofibroblast differentiation. The BET proteins Brd3 and Brd4 were shown to bind to the* NOX4* promoter and drive TGF-*β*-induced* NOX4* expression. Our data indicate a critical role of BET proteins in promoting redox imbalance and pulmonary myofibroblast activation and support BET bromodomain inhibitors as a potential therapy for fibrotic lung disease.

## 1. Introduction

Approximately two-thirds of patients with systemic sclerosis (SSc) develop some degree of interstitial lung disease (ILD) [1]. Pulmonary fibrosis, together with pulmonary hypertension, is the leading cause of death in SSc patients [2]. Idiopathic pulmonary fibrosis (IPF) is a progressive and inevitably fatal interstitial lung disease with a median survival since diagnosis of 3-5 years [3]. Both diseases are characterised by accumulation of extracellular matrix by an expanding population of myofibroblasts that show enhanced proliferation, migration, and resistance to apoptosis [4, 5].

Persistent TGF-*β* signaling is central in driving the myofibroblast phenotype in pulmonary fibrosis [6, 7]. Reactive oxygen species (ROS) are key mediators of TGF-*β* signaling in pulmonary fibroblasts [8, 9]. Intracellular ROS drive myofibroblast differentiation, and inhibiting ROS production ameliorates lung injury in bleomycin-treated mice [10, 11]. Increased levels of ROS [12, 13] and oxidative DNA damage [14] are observed in patients with SSc, and biomarkers of oxidative stress are elevated in IPF [15, 16], some of which negatively correlate with lung function [17, 18].

The prooxidant enzyme NADPH oxidase (NOX-4) and the antioxidant enzyme Mn-superoxide dismutase (MnSOD or SOD2) are central to intracellular ROS regulation. NOX4 reduces O_2_ into superoxide anion (O_2_^−^) and hydrogen peroxide (H_2_O_2_) [19]. NOX4 plays a critical role in TGF-*β*-induced ROS production, fibroblast-to-myofibroblast differentiation, and pulmonary fibrosis [20, 21]. SOD2 converts O_2_^−^ to H_2_O_2_ in the mitochondria and is an important antioxidant during oxidative stress [22]. The role of SOD2 in myofibroblast differentiation and in pulmonary fibrosis is unknown.

Bromodomain and extraterminal (BET) proteins have emerged as promising therapeutic targets for cancer [23], obesity, inflammation [23], and fibrosis [24, 25]. BET proteins (Brd2, Brd3, Brd4, and BrdT) are epigenetic readers, which bind to acetylated lysine residues on histone and nonhistone proteins via conserved bromodomains and regulate gene expression by recruiting transcriptional activators or repressors [23]. JQ1 is a small molecule inhibitor that inhibits the binding of BET protein bromodomains to acetylated lysines [23]. Recently, BET protein inhibition was shown to reduce lung myofibroblast activation and to have antifibrotic effects in an* in vivo* model of acute lung injury [24, 25]. However, the role of BET proteins in the regulation of intracellular redox state, in the context of lung fibrosis, has not been addressed.

We hypothesised that BET proteins drive redox imbalance and increased ROS production, contributing to myofibroblast differentiation. We therefore investigated the effect of JQ1 on redox balance, in TGF-*β*-stimulated pulmonary fibroblasts, by determining the expression of NOX4 and SOD2, the expression and activity of the antioxidant transcription factor NF-E2-related factor 2 (Nrf2), and intracellular ROS levels. The effect of JQ1 on the myofibroblast phenotype was studied by determining *α*SMA expression and myofibroblast contraction and proliferation. The role of specific BET protein isoforms in NOX4 gene expression was determined using chromatin immunoprecipitation (ChIP) and siRNA knock-down.

## 2. Materials and Methods

### 2.1. Reagents

JQ1(+) and its inactive enantiomer JQ1(-) were purchased from Cayman (Cambridge, UK), and TGF-*β*1 was purchased from R&D systems (Minneapolis, MN).

### 2.2. Fibroblast Cultures

**Figure 1:**
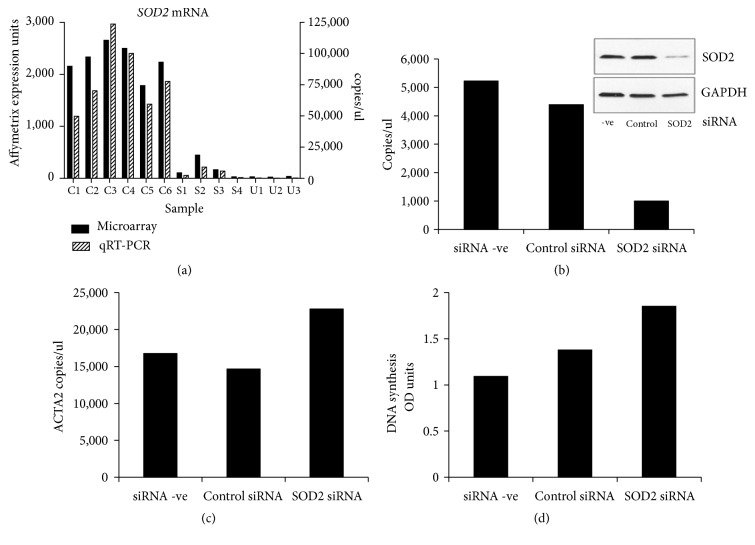
*Effect of SOD2 siRNA knock-down on αSMA expression and cell proliferation*. (a) Levels of* SOD2* mRNA in non-ILD control (C1-C6), SSc-ILD (S1-S4), and IPF (U1-U3) lung fibroblasts under basal serum-free conditions as determined by Affymetrix microarray analysis (black bars) were confirmed by RT-qPCR (grey bars). (b-d) Non-ILD control lung fibroblasts were either mock-transfected (siRNA –ve) or transfected with nontargeting siRNA (control siRNA), or* SOD2*-targeting siRNA for 72 h following which* SOD2* mRNA and protein (inset) (b) and (c)* ACTA2* mRNA expression levels were measured. (d) Proliferation induced by incubation with 3% FBS for 24 h was measured by BrdU incorporation. Data are shown as the mean of three independent experiments performed in two control cell lines ((b) and (c)) or in one control cell line (d), respectively.

Primary adult pulmonary fibroblasts were obtained from surgical lung resection samples from non-ILD donors (histologically normal periphery of resected lung cancer) and maintained as previously described [26, 27]. All tissue was obtained with appropriate consent and its use approved by the Ethics Committee of the Royal Brompton and Harefield National Health Service Trust. Subject details, fibroblast cultures, and experimental conditions used in the microarray study, from which data for Table 2 and Figure 1(a) were extracted, have been described previously [27]. Cells were plated for experiments at a density of 7000 cells/cm^2^ and were at ~90% confluence at the start of the experiment. For the proliferation assay, cells were stimulated at ~50% confluence.

### 2.3. RT-qPCR

Messenger RNA (mRNA) expression was determined using RT-qPCR as previously described [27, 28]. PCR primer details are provided in Table 1.

### 2.4. siRNA Silencing

Cells were transfected with 3 nM* Silencer*® Select negative control #1 siRNA or* SOD2, Brd3, *and* Brd4 *specific* Silencer*® Select siRNA (Ambion, Foster City, CA) for 72 h using Lipofectamine® RNAiMAX (Invitrogen) according to the manufacturer's instructions.

### 2.5. Western Blot Analysis

Whole cell extracts were separated by SDS-PAGE and transferred to a nitrocellulose membrane as previously described [28]. Proteins were detected using anti-SOD2 (Sigma-Aldrich, HPA001814; diluted 1:1000), anti-*α*-Smooth Muscle Actin (Sigma-Aldrich, A2547; diluted 1:1000), anti-beta Actin (Abcam, Cambridge, UK, ab8227), and mouse anti-GAPDH (Abcam, ab9484; diluted 1:2000) antibodies. Band intensities were quantified using ImageJ [29].

### 2.6. Proliferation Assay

Proliferation was assessed by measuring BrdU incorporation using the Cell Proliferation ELISA (Roche) according to the manufacturer's instructions.

### 2.7. Immunocytochemistry

Analysis was performed in 8-well chamber slides as previously described [28]. The primary antibody used was mouse anti-*α*-Smooth Muscle Actin (Sigma-Aldrich, A2547), used at a dilution of 1:100.

### 2.8. Collagen Contraction Assay

Collagen matrices were prepared as previously described [18], with some modifications. Briefly, fibroblasts (100,000/well) mixed with neutralised collagen (1.2mg/ml, Invitrogen) supplemented with 2.5*μ*M Mitomycin C (Sigma-Aldrich), 500 nM JQ1(-) or 500 nM JQ1(-), were cast into 24-well plates and allowed to polymerise for 1 h. Additional media (1mL/well) at indicated conditions were added, and the plates were incubated at 37°C. After 60 mins, 1 ng/ml TGF-*β*1 was added to some of the wells, as indicated, and the plates were incubated for further 24 h. The gels were released and allowed to contract for 4 h before fixing in 4% paraformaldehyde. The gel surface area was measured using ImageJ [29].

### 2.9. Determination of Intracellular ROS Levels

Intracellular ROS levels were determined by staining cells with the redox-sensitive fluorescent probe 6-carboxy-2′,7′-dichlorodihydrofluorescein diacetate (carboxy-H2DCFDA; Invitrogen) at 5*μ*M for 30 mins and measuring fluorescence using flow cytometry.

### 2.10. Promoter Reporter Assay

Nrf2 transcriptional activity was assessed using the Cignal Antioxidant Response Element (ARE) reporter assay (Qiagen, Crawley, UK) as described [28], with luminescence detected using the Dual-Luciferase® Reporter Assay System (Promega, Southampton, UK).

### 2.11. Chromatin Immunoprecipitation

Chromatin immunoprecipitation (ChIP) assays were performed using the Magna ChIP A/G kit (Merck Millipore, Billerica, MA) and 4 *μ*g of rabbit anti-Brd2 (A302-582A), anti-Brd3 (A302-368A), anti-Brd4 (A301-985A) (Bethyl Laboratories, Cambridge, UK), or Rabbit IgG control antibodies (Millipore, 12-370) as described previously [28]. DNA association was quantified by RT-qPCR (ΔΔ-Ct method) using the following primers: NOX4 forward, 5′-GCTTTAGTTTGGGAGTGGGA-3′, and reverse, 5′-GAAATTTGAGCCGGAAACAG-3′ [30]. DNA used was normalised to input DNA for each sample.

### 2.12. Statistical Analysis

Data are expressed as mean ± SEM or mean ± SD as indicated. Differences between groups were determined using repeated-measures analysis of variance (ANOVA) followed by a Dunnet's post hoc test. A value of p<0.05 was considered statistically significant. Statistical analysis was performed only in experiments performed on at least three individual cell lines (biological replicates).

## 3. Results

### 3.1. Dysregulation of* NOX4* and* SOD2* Gene Expression in Fibroblasts from Fibrotic Lung

In a previous microarray study, we reported increased* NOX4* mRNA expression in pulmonary fibroblasts from patients with SSc-ILD (16.9-fold) and IPF (26.4-fold). Here, we reassessed redox gene expression specifically and found that* SOD2* mRNA expression was markedly suppressed in both SSc-ILD (7.0-fold) and IPF (73.2-fold) fibroblasts, compared with non-ILD controls (Table 2, data extracted from [27]). The clinical characteristics of the subjects that donated fibroblasts used for microarray analysis were published previously [27].

### 3.2. *SOD2* Knock-Down Increases *α*SMA Expression and Inhibits Lung Fibroblast Proliferation

We confirmed the reduction of* SOD2* mRNA in fibrotic fibroblasts by RT-qPCR (Figure 1(a), gray bars) and compared it with that seen by microarray (Figure 1(a), black bars). Knock-down (KD) of* SOD2* expression with siRNA in lung fibroblasts attenuated SOD2 mRNA levels by approximately 75% (Figure 1(b)), with a corresponding reduction in* SOD2* protein levels (Figure 1(b), inset). SOD2 KD also led to an increasing trend in* ACTA2* mRNA expression (Figure 1(c)) and cell proliferation (Figure 1(d)).

### 3.3. JQ1 Inhibits TGF-*β*-Mediated *α*SMA Fibre Formation, Contractility, and Proliferation of Lung Fibroblasts

**Figure 2:**
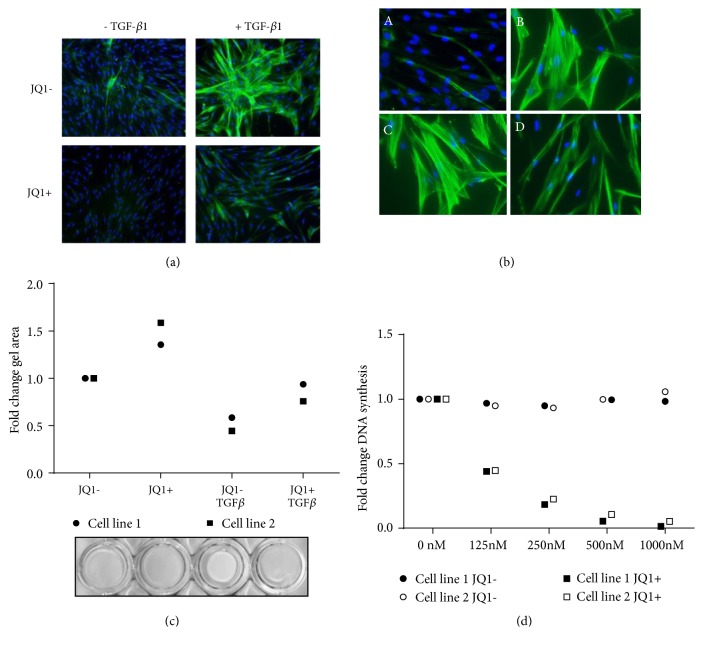
*Effect of JQ1 on TGF-β-induced myofibroblast phenotypic changes and proliferation*. (a) Non-ILD control fibroblasts were serum-starved for 24 h and pretreated with 500 nM JQ1 or JQ1(-) for 2 h before stimulation with TGF-*β*1 (1ng/ml) for 48 h. Cells were stained for *α*SMA (green) and nuclear DNA (DAPI/Blue). (b) Cells were cultured in the absence (picture A) or presence (picture B) of TGF-*β*1 for 48 h before being treated with 500 nM JQ1(-) (picture C) or JQ1(+) (picture D) for further 48 h. (c) Control cells were cast in collagen gels and treated with TGF-*β*1 and 500 nM JQ1 (+ or -) for 24 h. Gels were released and allowed to contract for 4 h, and the gel area was measured using ImageJ. Data points are mean from experiments in two different cell lines performed in triplicate and expressed as fold change compared to the JQ1- control. (d) Control fibroblasts were serum-starved and pretreated with JQ1 (+ or -) at the indicated concentrations before stimulation with 3% fetal bovine serum (FBS) for 24 h. Proliferation was determined by BrdU incorporation. Data points are mean from experiments in two different cell lines performed in triplicate and expressed as fold change compared to the JQ1- control.

TGF-*β* stimulation of non-ILD control lung fibroblasts for 48 h induced the formation of *α*SMA-containing stress fibres (Figure 2(a)). Pretreatment with JQ1(+) (500nM) attenuated basal and TGF-*β*-induced *α*SMA fibre formation. Importantly, established myofibroblast differentiation induced by 48 h TGF*β* stimulation (Figure 2(b), image B) was reversed by treatment with JQ1(+) (Figure 2(b), image D) but not by JQ1(-) (Figure 2(b), image C) for further 48 h.

As a functional readout of stress fibre content, we studied the effects of JQ1 on collagen gel contractility. JQ1(+) reduced both baseline and TGF-*β*-mediated gel contraction, as assessed by increased gel disc area (Figure 2(c)). JQ1(+) also inhibited proliferation induced by fetal bovine serum (FBS; 3%) (Figure 2(d)).

### 3.4. JQ1 Reduces *α*SMA Expression and Inhibits *β*-Actin Intracellular Distribution

**Figure 3:**
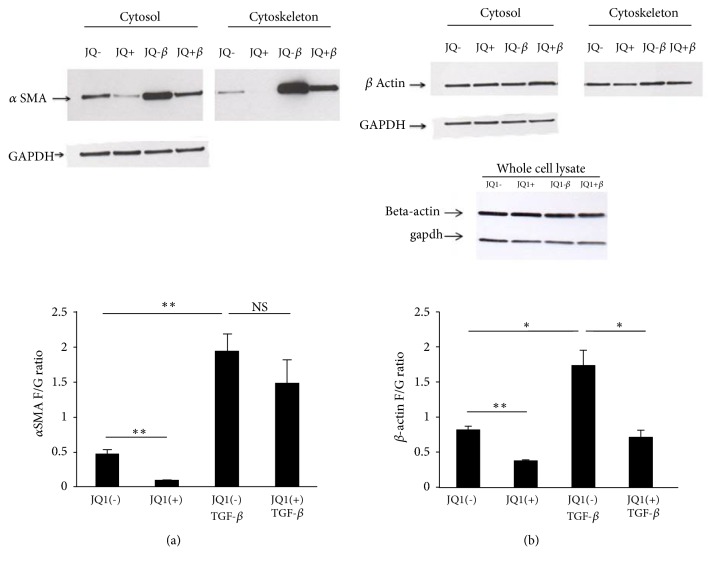
*Effect of JQ1 on αSMA and β-actin protein expression and intracellular distribution*. Protein expression of *α*SMA (a) and *β*-actin (b) was determined by Western blot analysis in non-ILD lung fibroblasts after 48 h in the presence or absence of TGF-*β*1 and JQ1(+) or JQ1(-). F-actin was separated from G-actin by differential solubility in Triton X-100. Both fractions had the same final volume, and equal volumes were run on SDS-PAGE gels. Total levels of *β*-actin were also analysed in whole cell extracts. Images are representative of three independent experiments. Signal intensity was measured using ImageJ. Bars represent mean ± SD from three independent non-ILD control cell lines. NS = nonsignificant. *∗*p<0.05 and *∗∗* p<0.01.

TGF-*β* enhanced both cytosolic and cytoskeletal *α*SMA protein expression (Figure 3(a), top panel) and caused an approximately 4-fold (p<0.01) shift in *α*SMA incorporation from globular cytosolic (G-actin) into fibrous cytoskeletal actin (F-actin) (Figure 3(a), graph). JQ1(+) markedly reduced *α*SMA protein expression in both fractions; however, it did not affect TGF-*β*-induced *α*SMA incorporation into fibres. Total *β*-actin protein expression was not significantly altered by either TGF-*β* or JQ1(+) (Figure 3(b)). However, the TGF-*β*-induced redistribution of *β*-actin from G-actin into F-actin was completely inhibited by JQ1(+) (p<0.05).

### 3.5. JQ1 Reverses the TGF*β*-Induced Imbalance in NOX4 and SOD2 Expression and Reduces Intracellular ROS Levels and ACTA2 Expression

**Figure 4:**
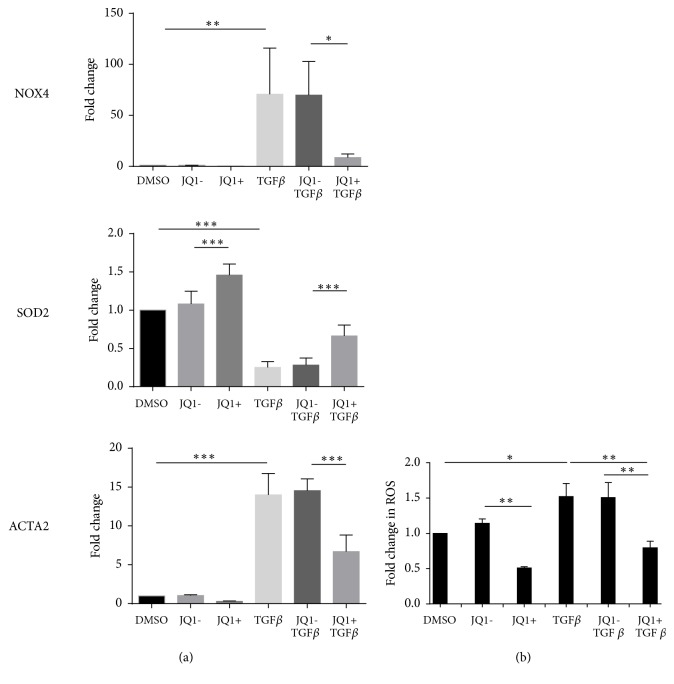
*Effect of JQ1 on TGF-β-induced NOX4, SOD2 and ACTA2 mRNA expression, and intracellular ROS*. Non-ILD control pulmonary fibroblasts were serum starved and pretreated with 500 nM JQ1(+) or JQ1(-) for 2 h before stimulation with TGF-*β*1 (1ng/ml) for further 24 h. (a) NOX4, SOD2, and ACTA2 mRNA was determined by RT-qPCR. (b) Intracellular ROS levels were determined by 6-carboxy-2′,7′-dichlorodihydrofluorescein diacetate staining. Data are expressed as fold change compared to the DMSO control and bars represent mean ± SD of independent experiments performed in three (a) and four (b) cell lines. *∗*p<0.05, *∗∗*p<0.01, and *∗∗∗*p<0.001.

JQ1(+) led to a reduction in* NOX4* and* ACTA2* mRNA compared to JQ1(-) after 24 h in non-ILD control pulmonary fibroblasts but did not reach statistical significance. In contrast, the expression of* SOD2* mRNA was significantly increased (1.45-fold, p<0.001) (Figure 4(a)). TGF-*β* increased* NOX4* (76-fold, p<0.01) and ACTA2 (14-fold, p<0.001) mRNA levels, while* SOD2* mRNA levels were significantly reduced (0.24-fold, p<0.001). JQ1(+) significantly attenuated TGF-*β*-induced* NOX4* (0.09-fold, p<0.05) and* ACTA2* (0.31-fold, p<0.001) expression and partially reversed the inhibition of* SOD2* mRNA levels by TGF-*β* (2.9-fold, p<0.01) (Figure 4(a)). In line with these findings, JQ1(+) significantly suppressed baseline (0.51-fold, p<0.01) and TGF-*β*-induced (0.53-fold, p<0.01) intracellular ROS in pulmonary fibroblasts (Figure 4(b)) There was no effect of JQ1(-) on ROS levels.

### 3.6. JQ1 Increases Nrf2 Activity

**Figure 5:**
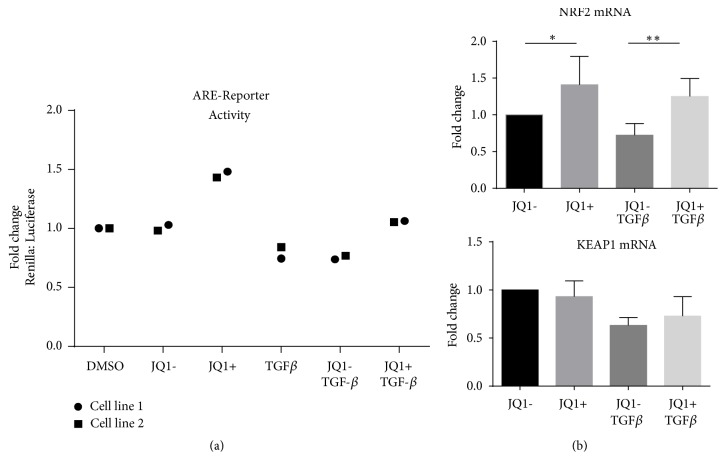
*Effect of JQ1 on Antioxidant Response Element- (ARE-) dependent transcription and Nrf2 and Keap1 mRNA expression*. (a) Non-ILD control fibroblasts were cotransfected for 48 hours with an inducible ARE-responsive Firefly-luciferase reporter construct and a Renilla-luciferase reference construct. Cells were serum-starved for 24 h and pretreated with 500 nM JQ1(+) or JQ1(-) for 2 h and then stimulated with TGF-*β*1 (1ng/ml) for 24 h. Bars represent fold change compared to DMSO control. Data points shown are two independent experiments performed in triplicate in two individual non-ILD control cell lines expressed as mean fold change compared to DMSO (vehicle control). (b) Cells were serum-starved for 24 h and pretreated with 500 nM JQ1(+) or JQ1(-) for 2 h before being stimulated with TGF-*β*1 (1ng/ml) for 24 h. NRF2 and KEAP1 mRNA levels were determined by RT-qPCR. Data are expressed as fold change compared to the JQ1- control. Bars represent mean ± SD of independent experiments in three individual non-ILD control lines. *∗*p<0.05 and *∗∗*p<0.01.

We have previously shown that BET proteins suppress Nrf2-mediated antioxidant gene transcription in airway smooth muscle cells (ASMCs) [28]. In the current study, JQ1(+) treatment of non-ILD lung fibroblasts led to an increasing trend in Antioxidant Response Element (ARE) promoter-driven gene transcription (Figure 5(a)) and a statistically significant increase in NRF2 mRNA both in absence and presence of TGF-*β* (Figure 5(b)), while JQ1(-) had no effect. JQ1(+) did not affect the mRNA expression of Keap1, the cytoplasmic inhibitor of Nrf2 (Figure 5(b)).

### 3.7. Brd3 and Brd4 Are Enriched at the NOX4 Promoter and Required for TGF*β*-Induced Gene Transcription

**Figure 6:**
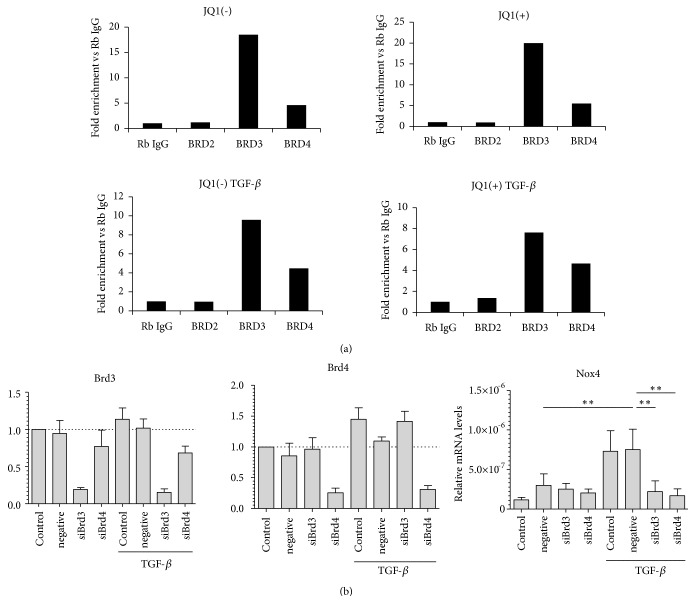
*Enrichment of Brd2, Brd3, and Brd4 at the NOX4 promoter and their role in TGFβ-induced Nox4 expression*. (a) Non-ILD control fibroblasts were starved for 24 h and pretreated with 500 nM JQ1(+) or JQ1(-) for 2 h before stimulation with TGF-*β*1 (1ng/ml) for further 4 h. Recruitment of Brd2, Brd3, and Brd4 to the NOX4 promoter was determined by ChIP. Data are expressed as fold enrichment compared to the IgG control. Data shown are the mean of two independent experiments performed in two control cell lines. (b) Non-ILD control fibroblasts were either mock-transfected (Control) or transfected with nonspecific siRNA control (negative) or with specific siRNAs to* BRD3* (siBrd3) or* BRD4* (siBrd4). Cells were stimulated with TGF-*β*1 for 24 h and the expressions of* BRD3*,* BRD4,* or* NOX4* mRNA levels were determined by RT-qPCR. The data is presented as the mean ± SEM of independent experiments on four individual cell lines. *∗∗*p<0.01 and *∗∗∗*p<0.001.

ChIP assays in non-ILD fibroblasts from two subjects in two independent experiments demonstrated that Brd3 and Brd4, but not Brd2, bind to the NOX4 promoter (Figure 6(a)). Furthermore, Brd3 or Brd4 siRNA knock-down, which efficiently reduced Brd3 and Brd4 mRNA, respectively, led to inhibition of TGF-*β*-induced* NOX4* mRNA expression, suggesting a role of both these proteins in driving* NOX4* gene expression (Figure 6(b)).

## 4. Discussion

We show that the small molecule BET protein inhibitor JQ1 reduces basal and TGF-*β*-induced NOX4 expression and ROS production. JQ1 also increases the expression of the antioxidant gene SOD2 and the activity of the cytoprotective transcription factor Nrf2 and prevents the inhibition of SOD2 expression by TGF-*β*. At the same time, JQ1 prevents and reverses TGF-*β*-mediated myofibroblast differentiation. The suppressive effect of BET protein inhibition on myofibroblast differentiation may be mediated, at least partly, through restoring redox balance via SOD2 regulation, as SOD2 depletion was found to increase *α*SMA expression and fibroblast proliferation.

The important role of oxidative stress in pulmonary fibrosis has directed therapeutic efforts towards inhibiting ROS generation and enhancing antioxidant capacity [31]. Several therapies involving antioxidant compounds, such as* N*-acetylcysteine (NAC), have been tested and while promising in preclinical models have ultimately lacked clinical efficacy. Targeting a primary source of intracellular ROS by inhibiting NOX4 activity has lately been a strong focus in antifibrotic drug development. However, there are currently no clinically available, selective NOX4 inhibitors [31].

NOX4 overexpression has been shown to be fundamental in maintaining a myofibroblast phenotype [20]. Here we demonstrate that BET proteins, and specifically Brd3 and Brd4, drive NOX4 expression in pulmonary fibroblasts. Our findings are in line with recent evidence demonstrating a role of Brd4 in TGF-*β*-induced NOX4 expression in skin and renal fibroblasts [32, 33]. Inhibition of NOX4 expression by JQ1 was accompanied by a reduction in ROS levels,* ACTA2* gene expression, stress fibre formation, myofibroblast contraction, and proliferation.* NOX4 *is one of the early key genes involved in the initiation of a TGF-*β*-stimulated fibrotic response [8, 20]. Therefore, the antifibrotic effects of JQ1 may be mediated, at least partly, through inhibition of NOX4 expression. Importantly, our finding that Brd3 and Brd4, but not Brd2, are involved in NOX4 regulation by TGF-*β* suggests that a degree of selectivity may be possible when considering BET inhibitors as possible therapeutic agents in pulmonary fibrosis. However, a study by Tang* et al.*, also performed in human lung fibroblasts, reported that Brd2 and Brd4, but not Brd3, mediate TGF-*β*-mediated *α*-SMA expression and myofibroblast activation [24]. This apparent disparity is hard to explain at this point; however, it could suggest that the specificity of BET protein isoforms recruitment to specific gene promoters may be context-specific and dependent on timing and culture conditions. In addition, detailed studies elucidating the roles of specific BET proteins are technically challenging and were relatively limited in both these studies. For example, Tang* et al.* focused only on Brd4 to show direct binding to promoters of fibrosis-related genes, *α*SMA (*ACTA2*), IL6, and PAI-1 (SERPINE1), whereas our study investigated binding of all three BET protein isoforms on the NOX4 promoter. More in-depth investigations will be necessary to delineate the roles of specific BET proteins in progressive fibrosis with a view to targeting them for treatment.

TGF-*β*-mediated upregulation of NOX4 was accompanied by a reduction in SOD2 expression, in line with our previous findings in ASMC [34]. Attenuated SOD2 expression may contribute to the profibrotic effect of TGF-*β*, as we show here that knock-down of* SOD2 *expression results in increased* ACTA2* expression and fibroblast proliferation. Reduced SOD2 expression in pulmonary arterial smooth muscle cells of patients with pulmonary arterial hypertension (PAH) has been associated with a phenotype of increased proliferation and lower apoptosis [22]. Downregulation of SOD2 has also been shown to mediate TGF-*β*-induced epithelial-mesenchymal transition, a profibrotic mechanism, in renal epithelial cells [35]. These findings suggest that the antifibrotic effect of JQ1 may, at least partly, occur through the upregulation of SOD2.

We observed that, during the TGF-*β*-induced fibrotic state, there was a trend towards reduced expression and activity of Nrf2, a key mediator of cellular antioxidant protection [36]. Furthermore, on reexamination of our previous microarray data, we found that the Nrf2 expression was significantly lower in pulmonary fibroblasts from IPF (fold change -1.77, p=0.0096) and from SSc-ILD (fold change -1.52, p=0.02) lung compared with non-ILD control lung [27]. Reduced Nrf2 gene expression has been reported previously in IPF pulmonary fibroblasts compared to controls, and Nrf2 knock-down has been shown to induce oxidative stress and myofibroblast differentiation [37]. JQ1 increased Nrf2 expression and activity in both absence and presence of TGF-*β*, indicating that BET protein inhibition can increase antioxidant protection in activated fibroblasts. Our data are consistent with previous findings by our group showing that BET proteins interact with Nrf2 and inhibit ARE-dependent antioxidant gene transcription in ASMC [28]. Studies in WI-38 cells have also reported that BRD4 knock-down leads to increased Nrf2 nuclear accumulation and activity under oxidative stress [38]. A more in-depth investigation, including a study of the interaction of Keap1 with actin fibres, known to be essential for Nrf2 inactivation, is required to better understand the regulation of Nrf2 activity by BET proteins in pulmonary fibroblasts [39].

Our data highlight an important new molecular mechanism regulating redox balance and myofibroblastic differentiation. Future studies will need to confirm this mechanism in fibroblasts from patients with pulmonary fibrosis and in animal models of pulmonary fibrosis to firmly establish a role of BET proteins in regulating redox gene expression and ROS levels during fibrosis.

In summary, our data suggest that BET protein inhibition restores redox balance through its effects on NOX4/SOD2 expression and Nrf2 activity, leading to the reprogramming/dedifferentiation of myofibroblasts. Our findings highlight BET protein inhibitors as potential antifibrotic therapeutic agents. Studies are now urgently needed to assess the suitability of these compounds for pulmonary fibrosis.

## Figures and Tables

**Table 1:** Primers used for RT-qPCR analysis.

Gene	Primer sequence
*NOX4*	forward 5′-CTGCTGACGTTGCATGTTTC-3′

	reverse 5′-CGGGAGGGTGGGTATCTAA-3′

*SOD2*	forward 5′-CACCAGCACTAGCAGCATGT-3′

	reverse 5′-GAGCCCAGATACCCCAAAAC-3′

*ACTA2*	forward 5′-CCCTGAAGTACCCGATAGAACA-3′

	reverse 5′-GGCAACACGAAGCTCATTG-3′

*YWHAZ*	forward 5′-ACTTTTGGTACATTGTGGCTTCAA-3′

	reverse 5′-CCGCCAGGACAAACCAGTAT-3′

*HPRT1*	forward 5′-TGACACTGGCAAAACAATGCA-3′

	reverse 5′-GGTCCTTTTCACCAGCAAGCT-3′

*BRD3*	Applied Biosystems TaqMan probe Hs00201284_m1

*BRD4*	Applied Biosystems TaqMan probe Hs04188087_m1

*NRF2 (NFE2L2)*	QuantiTect Primer Assay QT00027384

*KEAP1*	QuantiTect Primer Assay QT00080220

**Table 2:** *NOX4* and *SOD2* gene expression data extracted from microarray analysis.

Gene	Control (n=10)	SSc-ILD (n=8)	Fold change	p value	IPF (n=3)	Fold change	p value
*NOX4*	12.3	206.6	16.9	0.012	323.6	26.4	0.016

*SOD2*	2180.4	311.0	-7.0	<1x10^−6^	29.8	-73.2	<1x10^−6^

Basal serum-free gene expression in non-ILD control, SSc-ILD, and IPF lung fibroblasts was determined by Affymetrix microarray analysis [27]. Mean expression levels are given in Affymetrix units. Fold changes for SSc-ILD and IPF fibroblasts, and p values adjusted for multiple comparisons, are relative to the expression levels in non-ILD control cells.

## Data Availability

All the data used to support the findings of this study are included within the article.
